# Effect of microplastics on nasal and intestinal microbiota of the high-exposure population

**DOI:** 10.3389/fpubh.2022.1005535

**Published:** 2022-10-28

**Authors:** Xiyu Zhang, Heting Wang, Sihan Peng, Jian Kang, Ziyan Xie, Ruobing Tang, Yiqian Xing, Yuchi He, Haipo Yuan, Chunguang Xie, Ya Liu

**Affiliations:** ^1^TCM Regulating Metabolic Diseases Key Laboratory of Sichuan Province, Hospital of Chengdu University of Traditional Chinese Medicine, Chengdu, China; ^2^Department of Traditional Chinese Medicine, Sichuan Provincial People's Hospital, University of Electronic Science and Technology of China, Chengdu, China; ^3^School of Clinical Medicine, Chengdu University of Traditional Chinese Medicine, Chengdu, China; ^4^Proctology Department, University of Traditional Chinese Medicine, Chengdu, China

**Keywords:** microplastics, environment, nasal microbiota, intestinal microbiota, 16s rDNA sequencing, laser infrared imaging

## Abstract

**Background:**

Microplastic has become a growing environmental problem. A balanced microbial environment is an important factor in human health. This study is the first observational cross-sectional study focusing on the effects of microplastics on the nasal and gut microbiota in a highly exposed population.

**Methods:**

We recruited 20 subjects from a Plastic Factory (microplastics high-exposure area) and the other 20 from Huanhuaxi Park (microplastics low-exposure area) in Chengdu, China. We performed the microplastic analysis of soil, air, and intestinal secretions by laser infrared imaging, and microbiological analysis of nasal and intestinal secretions by 16S rDNA sequencing.

**Results:**

The result shows that the detected points of microplastics in the environment of the high-exposure area were significantly more than in the low-exposure area. Polyurethane was the main microplastic component detected. The microplastic content of intestinal secretions in the high-exposure group was significantly higher than in the low-exposure group. Specifically, the contents of polyurethane, silicone resin, ethylene-vinyl acetate copolymer, and polyethylene in the high-exposure group were significantly higher than in the low-exposure group. Moreover, high exposure may increase the abundance of nasal microbiotas, which are positively associated with respiratory tract diseases, such as *Klebsiella* and *Helicobacter*, and reduce the abundance of those beneficial ones, such as *Bacteroides*. Simultaneously, it may increase the abundance of intestinal microbiotas, which are positively associated with digestive tract diseases, such as *Bifidobacterium, Streptococcus*, and *Sphingomonas*, and reduce the abundance of intestinal microbiotas, which are beneficial for health, such as *Ruminococcus Torquesgroup, Dorea, Fusobacterium*, and *Coprococcus*. A combined analysis revealed that high exposure to microplastics may not only lead to alterations in dominant intestinal and nasal microbiotas but also change the symbiotic relationship between intestinal and nasal microbiotas.

**Conclusion:**

The results innovatively revealed how microplastics can affect the intestinal and nasal microecosystems.

**Clinical trial registration:**

ChiCTR2100049480 on August 2, 2021.

## Introduction

Microplastic initially referred to as the tiny plastic particles present in the ocean (1). Microplastics mostly come from human daily activities, whose sizes are usually within 5 mm (2). These plastic polymers can accumulate in landfills and the natural environment as they cannot be biodegraded (3). Plastic waste production worldwide is expected to increase by 155–265 million tons per year by 2060 (4). Microplastics are present in every possible place that people could ever imagine, including sea, air, soil, and even the South Pole (5–9).

Studies have shown that the microplastics from the air would mostly gather in the nasal cavity, whereas the microplastics from the food would enrich the intestine (10). The human biomonitoring study reported the presence of plastic in lung tissues, which indicates that microplastics from the air can deposit and accumulate in the lung (11). Studies indicated that the respiratory diseases of workers from synthetic material factories who are exposed to airborne microplastics have something to do with their careers (12). Long-term exposure to microplastics can cause lung diseases, including asthma and pneumoconiosis (13–15). Furthermore, ingested microplastics can accumulate in the intestine, which would injure intestinal epithelial cells, disrupt the intestinal barrier, and produce enterotoxin effects (16–19). A balanced microbial environment is an important factor in human health. Microplastics can lead to diseases through food and air entering into the intestine and lungs. Some studies have found that exposure to microplastics for a long time could make a difference to microbiota *in vivo*, thus, causing diseases in multiple systems (20–22).

The study was expected to find out what impact microplastics could have on nasal and intestinal microbiota of the high-exposure population, such as workers in the plastic factory. The results of these two population comparisons provided interesting and important insights into the impact of microplastics on human health.

## Materials and methods

### Ethical approvals and consent to participate

The Medical Ethics Committee of the Hospital of Chengdu University of Traditional Chinese Medicine has approved this trial and the approval number is 2021KL-065. The informed consent has been signed by all subjects. The study has been implemented in accordance with the guidelines of the Declaration of Helsinki.

### Study subject

We recruited 20 subjects who worked and lived in this area from a plastics factory (microplastics high-exposure area, HM) and the other 20 subjects who worked and lived in this area within 1 km of the Huanhuaxi Park (microplastics low-exposure area, LM). Located in the center of Chengdu, China, Huanhuaxi Park has indisputably good air quality since it has the largest forest and wetland (32.32 hectares) in Chengdu. In addition, to ensure the quality of the urban environment, the Chengdu government has not set up plastic factories and other industries that are prone to pollution in the surrounding area. Volunteers who met the criteria below would officially be a subject of this study. The inclusion criteria were as follows: (1) Participants who voluntarily participate in this study and sign an informed consent form; (2) Males and females age 18 through 65; (3) No nasal diseases such as a nasal tumor, congenital malformation, structural abnormality, and respiratory diseases; (4) No organic digestive system diseases such as peptic ulcer, inflammatory bowel diseases, gastrointestinal tumor; (5) No serious primary diseases in the cardiovascular, digestive, urinary, and hematopoietic systems; (6) Participants stay in the area for more than 20 hours a day, which should last for more than 3 years, with less than 7 days of absence every year. The exclusion criteria were as follows: (1) History of systemic or nasal use of antibiotics, antifungals, hormones, and other medications affecting the microecology of the flora within 3 months; (2) History of systemic or nasal use of probiotics/probiotic products (including medications, yogurt, and beverages) within 3 months; (3) No active upper respiratory system infection findings within 3 months; (4) No active dysbiotic symptoms conditions (diarrhea, gas, and bloating) within 3 months; (5) Obviously mental disorders; (6) Long history of smoking; (7) Pregnancy and lactation.

### Sampling and sample processing

#### Collecting samples of intestinal secretions

Subjects would receive necessary training so that they could collect the intestinal secretions themselves. The samples of high and low-exposure groups would be named IHM and ILM, respectively. We would give them cryopreservation boxes, sterile feces collectors, and sterile glass vials at the beginning. Specimens in the feces collectors would be used for 16S rDNA sequencing, whereas specimens in the glass vials would be used for microplastic composition analysis.

The samples would be stored in the cryopreservation box. Members of our group would be responsible for transferring those samples. We would place sterile feces collectors in liquid nitrogen for 4 h, and then transfer them to −80°C for storage pending testing. Sterile glass vials would be stored at 4°C for testing.

#### Collecting samples of nasal secretions

Members of the study would be trained to perform sample collections. Nasal samples would be collected with the help of rhinoscopy. We would place the samples in liquid nitrogen for 4 h and then transfer them to −80°C for storage. The samples of high and low-exposure groups would be named as NHM and NLM, respectively.

#### Collecting samples from the environment

We would collect environmental samples from Huanhuaxi Park and the plastic factory. Samples would be collected at five spots, including the east, west, south, north, and center of the selected area. The topsoil (10 cm) at five sampling points will be collected using a stainless steel sampling shovel. Those samples will be mixed uniformly to be one composite sample and wrapped in aluminum foil in a sampling bag. Air samples were collected through a filter membrane hanging on a pole with a height of 1.5 m (adult breathing height), with three filter membranes hanging on each pole. After 48 h, the filter membranes would be collected and placed in glass containers for detection (Supplementary Figure 1) (23).

### Microbiota analysis by 16S rDNA gene sequencing

Microbial genomic DNA would be isolated using a DNeasy PowerSoil kit (Qiagen, Hilden, Germany) following the manufacturer's instructions. The Amplicon quality would be visualized using gel electrophoresis. The PCR products would be purified with the Agencourt AMPure XP beads (Beckman Coulter Co., USA) and quantified using the Qubit dsDNA assay kit. Sequencing would be performed on an Illumina NovaSeq6000 with two paired-end read cycles of 250 bases each (Illumina Inc., San Diego, CA; OE Biotech Company; Shanghai, China). The primers are 343F TACGGRAGGCAGCAG; 798R AGGGTATCTAATCCT (24). Paired-end reads were then preprocessed using the Cutadapt software to detect and cut off the adapter. After trimming, paired-end reads would be filtering low-quality sequences, denoise, merge, and detect and cut off the chimera reads using DADA2 with the default parameters of QIIME2 (25, 26). We unified the sequencing depth among different samples at the minimum sequencing depth level. We rarefied each sample to 41941 sequences before calculating the diversity indices. At last, the software output the representative reads and the Amplicon Sequence Variant (ASV) abundance table. The representative read of each ASV would be selected using the QIIME 2 package. All representative reads would be annotated and blasted against Silva database Version 138 (or Unite) using q2-feature-classifier with the default parameters. The 16S rRNA gene amplicon sequencing and analysis would be conducted by the OE Biotech Co., Ltd. (Shanghai, China).

### Microplastics analysis by laser infrared imaging

#### Analysis of microplastics from intestinal secretions

Excess concentrated nitric acid (68%) was added to intestinal secretion samples to digest proteins. The solution was neutralized using 10 wt% NaOH. For large particles with a diameter size >500 μm, a large-aperture filter for interception and vacuum filtration was employed. After rinsing three times with hydrogen peroxide and ethanol, the resulting filter membrane was immersed in ethanol solution for sonication to disperse the substance on it in the solution. After removing the filter membrane in the solution and washing it with ethanol for three times, the ethanol solution was concentrated, then dropped on a high-reflection glass, and tested by laser infrared imaging (8700 LDIR, Agilent Technologies Co., Ltd, USA) after the ethanol was completely volatilized. The microplastic spectral library was established with the setting mode of matching degree > 0.65 and the equivalent diameter range of 20–500 μm. Matching degree: based on the similarity between the infrared spectrum of the particle sample and the standard spectrum in the spectrum library, the maximum is 1. Equivalent diameter: the irregular area of a particle sample identified is equivalent to the size of a circular diameter.

### Analysis of microplastics from soil

Prepare 1.7–1.8 kg/L ZnCl_2_ (high-grade purity) solution. Put 30 g soil sample into a 100 mL beaker, and add 60 mL of zinc chloride solution, stir for 2 min, and stand for 12 h. The suspension would be transferred to another beaker and 60 mL of 30% H_2_O_2_ would be added to remove organic matter. The solution was stranded for 24 h after sufficient stirring to allow the hydrogen peroxide to react adequately with organic matter. Vacuum filtration was employed on the hydrogen peroxide-treated solution. The obtained filter membrane was immersed in ethanol solution for sonication to disperse the substance on it in the solution. The subsequent steps were the same as intestinal secretions.

### Analysis of microplastics from air particles

The filter membrane collecting outdoor air particles was immersed in ethanol solution for sonication to disperse the substance on it in the solution. The subsequent steps were the same as intestinal secretions.

### Statistical analysis

SPSS 24.0 (IBM, Armonk, NY, USA) and Prism 6.0 (GraphPad, San Diego, CA, USA) would be used for data analysis. Continuous data are the mean ± SD. The significant differences would be analyzed using the one-way analysis of variance (ANOVA) followed by Tukey's multiple comparisons in multiple groups. The permutational multivariate analysis of variance analyses of PCoA would be performed so that we could observe the overall distribution among groups. Correlations would be analyzed by using Spearman's correlation analysis. Differences would be significant if *P* < 0.05.

## Results

### Demographics

There were 40 subjects in total, 20 for the microplastics high-exposure group and the other 20 for the low-exposure group. The median age was 44.5 years in the high-exposure group and 38.5 years in the low-exposure group, with no difference in age between the two groups (*P* > 0.05). The median height was 166 cm in the high-exposure group and 160 cm in the low-exposure group, with no difference in height between the two groups (*P* > 0.05). The mean body weight was 60.4 ± 8.1 kg in the high-exposure group and 60.1 ± 9.3 kg in the low exposure group, with no difference in body weight between the two groups (*P* > 0.05). The data of subjects are shown in Table 1.

**Table 1:** Demographics of the study groups.

**Characteristics/parameters**	**HM (*n* = 20)**	**LM (*n* = 20)**	***p***
**Sex (%)**			
Male	11(55)	5(25)	
Female	9(45)	15(75)	
Age, median (IQR) (years)	44.5 (37.75–49.75)	38.5 (26.5–57)	0.253
Height, median (IQR) (cm)	166 (156.5–170)	160 (158.5–168.7)	0.602
Body weight (kg)	60.4 ± 8.1	60.1 ± 9.3	0.936

### Analysis of microplastics from soil

**Figure 1:**
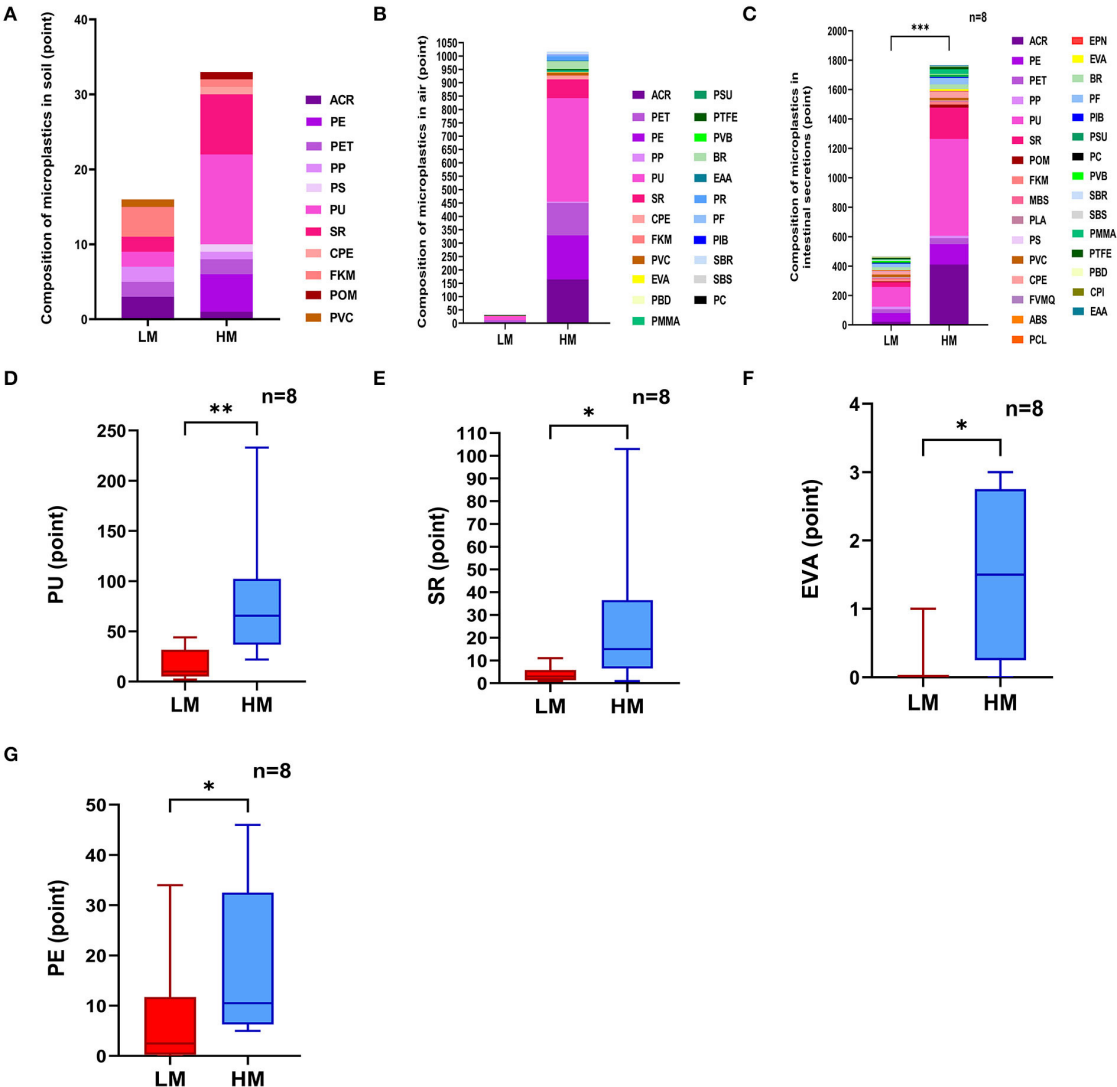
Analysis of microplastics. **(A)** Analysis of microplastics from soil. **(B)** Analysis of microplastics from air particles. **(C–G)** Analysis of microplastics from intestinal secretions. Acrylic resin, ACR; Polyethylene, PE; Polyethylene terephthalate, PET; Polypropylene, PP; Polystyrene, PS; Polyurethane, PU; Silicon resin, SR; Chlorinated polyethylene, CPE; Fluoro rubber, FKM; Polyoxymethylene, POM; Polyvinylchloride, PVC; Ethylene-vinyl acetate, EVA; Polybutadiene, PBD; Polymethyl methacrylate, PMMA; Polysulfone, PSU; Polytetrafluoroethylene, PTFE; Polyvinyl Butyral, PVB; Butadiene rubber, BR; Ethylene-acrylic acid copolymer, EAA; Petroleum resin, PR; Phenolic resin, PF; Polyisobutylene, PIB; Properties, SBR; Styrene-butadiene-styrene copolymer, SBS; Polycarbonate, PC; Polyoxymethylene, POM; Methylmethacrylate-butadiene-styrene, MBS; Polylactic acid, PLA; Fluorosilicone rubber, FVMQ; Acrylonitrile-butadiene-styrene, ABS; Polycaprolactone, PCL; Novolac epoxy resin, EPN; Polysulfone, PSU; Polybutadiene, PBD; Chlorinated Polyisoprene, CPI; Ethylene-acrylic acid copolymer, EAA. *P < 0.05, **P < 0.01.

Soil samples from both areas detected 11 different kinds of microplastics in total, with equivalent diameters ranging from 20 to 500 μm. The detected points of microplastics were 33 in the high-exposure group and 16 in the low-exposure group. Among them, polyurethane (PU) was the main microplastic detected, accounting for 36.36% in the high-exposure group and 12.5% in the low-exposure group. Silicon resin (SR) accounted for 24.24% of the high-exposure group and 12.5% of the low-exposure group (Figure 1A). The Laser infrared imaging diagrams of the two groups are shown in Supplementary Figures 2A,B.

### Analysis of microplastics from air particles

Air samples from both areas detected 23 different kinds of microplastics in total, with equivalent diameters ranging from 20 to 500 μm. The detected points of microplastics were 1,017 in the high-exposure group and 30 in the low-exposure group. Polyurethane (PU) was the main microplastic detected, accounting for 38.05% in the high-exposure group and 50% in the low-exposure group. Acrylic resin (ACR) accounted for 16.22% of the high-exposure group and 6.66% of the low-exposure group (Figure 1B). The Laser infrared imaging diagrams of the two groups are shown in Supplementary Figures 2C,D.

### Analysis of microplastics from intestinal secretions

Intestinal secretion samples from both groups detected 31 different kinds of microplastics, with equivalent diameters ranging from 20 to 500 μm. The microplastic content of intestinal secretions from the high-exposure group was significantly higher than the low-exposure group (*P* < 0.001) (Figure 1C). Polyurethane (PU) was the main microplastic detected, accounting for 37.30% in the high-exposure group and 29.09% in the low-exposure group. Silicon resin (SR) accounted for 12% of the high-exposure group and 6.9% of the low-exposure group. Specifically, the contents of polyurethane (PU), silicone resin (SR), ethylene-vinyl acetate copolymer (EVA), and polyethylene (PE) of intestinal secretions from the high-exposure group were significantly higher than the low-exposure group (*P* < 0.05 or *P* < 0.01) (Figures 1D–G). The laser infrared imaging diagrams of the two groups are shown in Supplementary Figures 2E,F.

### Analysis of nasal and intestinal microbiota

**Figure 2:**
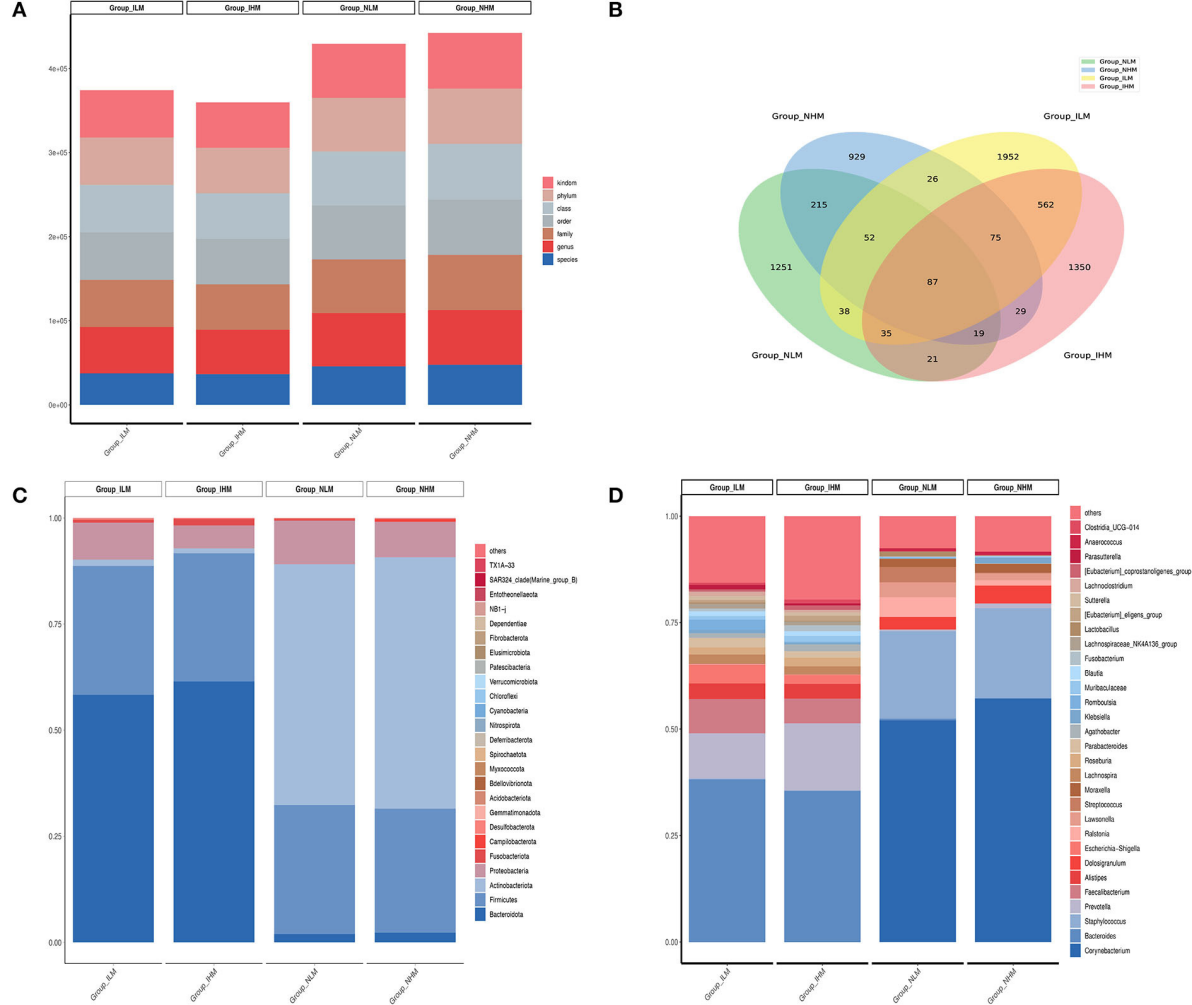
Histogram of abundance-ranked. **(A)** The total amount of valid tags obtained from the four groups. **(B)** A Venn diagram. **(C,D)** The histogram of abundance-ranked top 30 microbes.

The total amount of raw reads obtained from the detection of 80 samples were distributed from 78,029 to 81,706. The total amount of valid tags obtained from the four groups for analysis was distributed from 41,941 to 69,996 (Figure 2A). A Venn diagram was used to compare the similarity and specificity of the distribution of the four group microbiotas (Figure 2B). There were 87 shared ASV in the four groups, 373 shared ASV in the NLM and NHM groups, 759 shared ASV in the ILM and IHM groups, 1,251 specific ASV in the NLM group, 929 specific ASV in the NHM group, 1,952 specific ASV in the ILM group, and 1,350 specific ASV in the IHM group.

At the phylum level, the histogram of abundance-ranked top 30 microbes is shown in Figure 2C. *Bacteroidota* (58.3 and 61.4%) and *Firmicutes* (30.3 and 30.2%) were the dominant flora of ILM and IHM groups. *Actinobacteriota* (56.7 and 59.2%) and *Firmicutes* (30.3 and 29.2%) were the dominant flora of NLM and NHM groups. Among them, nasal and intestinal microbiota in the low-exposure group, such as *Bacteroidota* (1.96 and 58.3%) and *Actinobacteriota* (56.7 and 1.4%), were significantly different. Nasal and intestinal microbiota in the high-exposure group, such as *Bacteroidota* (2.3 and 61.4%) and *Actinobacteriota* (59.2 and 1.1%), were also significantly different.

At the genus level, the histogram of abundance-ranked top 30 microbes is shown in Figure 2D. *Bacteroides* (38.0 and 35.3%) and *Prevotella* (10.6 and 15.7%) were the dominant flora of ILM and IHM groups. *Corynebacterium* (52.0 and 57.0%) and *Staphylococcus* (20.5 and 21.0%) were the dominant flora of NLM and NHM groups. Among them, nasal and intestinal microbiota in the low-exposure group, such as *Corynebacterium* (52.0 and 0.1%), *Bacteroides* (0.3 and 38.0%), and *Staphylococcus* (20.5 and 0.2%), were significantly different. Nasal and intestinal microbiota in the high-exposure group, such as *Corynebacterium* (57.0 and 0.1%) and *Bacteroides* (0.1 and 35.3%) were also significantly different.

**Figure 3:**
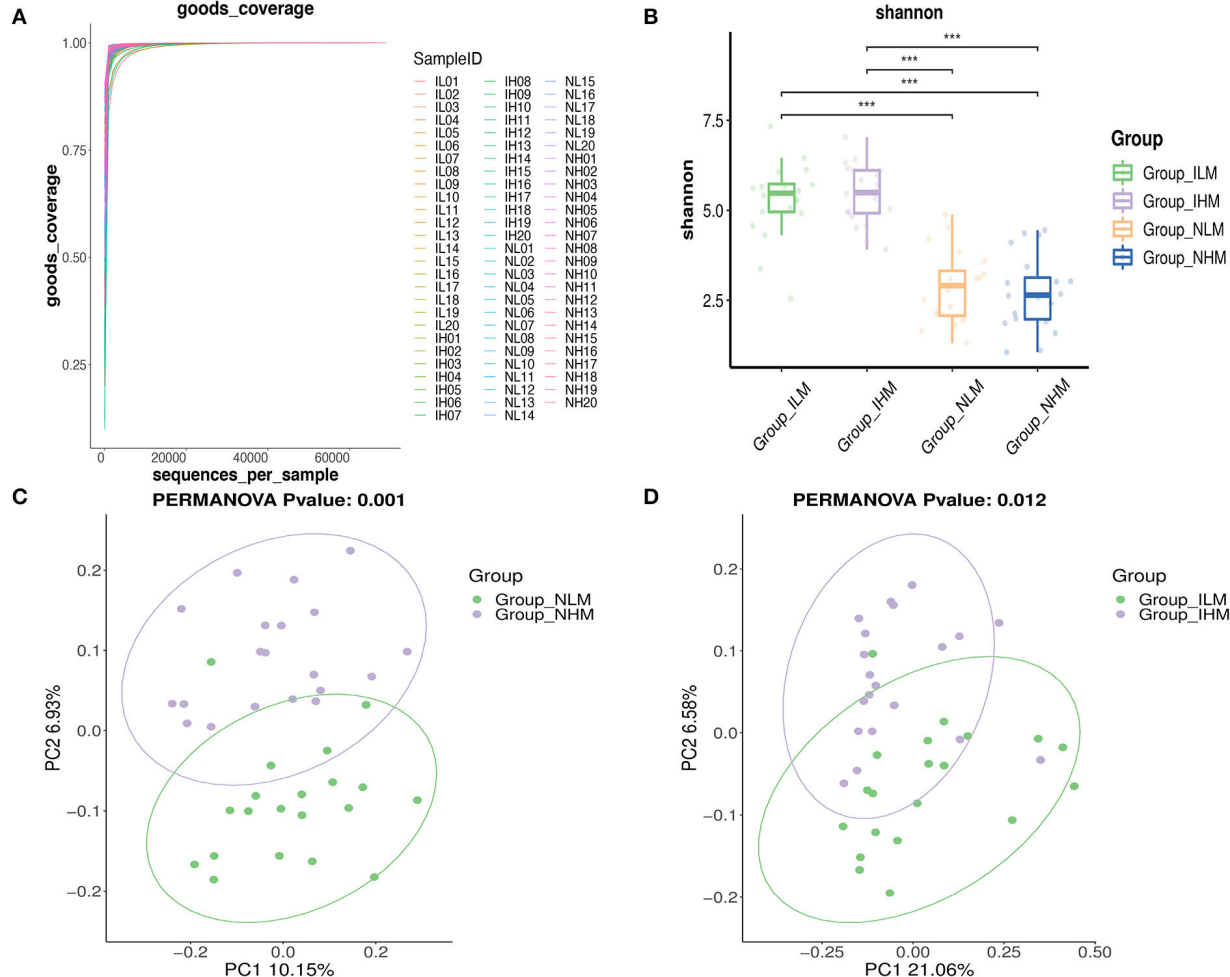
Alpha and beta diversity analysis. **(A)** The Good's Coverage. **(B)** The Shannon-Weaver index. **(C,D)** Each point in the PCoA represents a sample. Points in the same color are in the same group. The closer the points in the same group are, and the further the points to other groups are, the better the grouping effect is. ***P < 0.001.

Alpha diversity is used to measure the richness of species in a community. Alpha diversity is a comprehensive indicator of species and richness and evenness. The Good's Coverage reflects the sequencing depth, the closer it is to 1, the wider range of species in the samples the sequencing depth covers (Figure 3A). The Shannon-Weaver reflects species richness and evenness of distribution. The higher the species diversity and the more uniform the species distribution are, the higher the value of the Shannon-Weaver index (27). As shown in Figure 3B, there was no significant difference in the richness estimator and diversity index of nasal and intestinal microbiota between the two groups. Whereas, there was a significant difference in the richness estimator and diversity index between nasal and intestinal microbiota in low-exposure and high-exposure groups.

To assess the overall bacterial composition for four groups, we performed Principal Coordinates Analysis (PCoA) in beta diversity analysis. As shown in Figures 3C,D, the bacterial communities between the low-exposure group and the high-exposure group both in the intestine and nasal showed different patterns.

**Figure 4:**
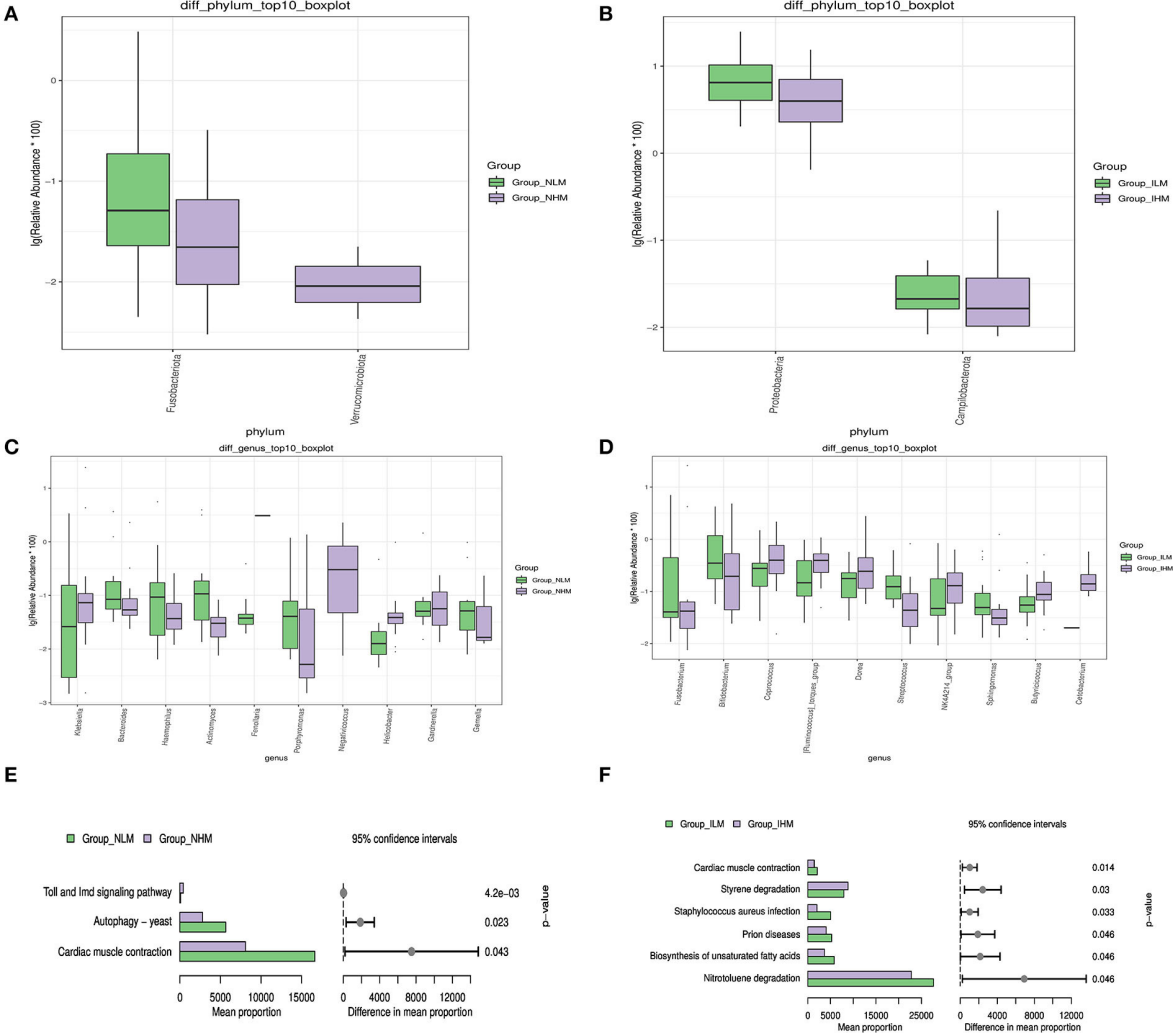
Microbiological multivariate statistical analysis. **(A–D)** The Wilcoxon analysis. **(E,F)** Prediction of gene function of known microbes.

Microbiological multivariate statistical analysis would be used to assess significant differences between the two groups at every level. We selected species that were abundance-ranked top 10 to perform the Wilcoxon analysis to compare the differences in dominant species. At the phylum level, the relative abundances of *Fusobacteriota* in the NHM group were significantly lower than in the NLM group (*P* < 0.05) (Figure 4A). The relative abundances of *Proteobacteria* and *Campilobacterota* in the IHM group were significantly lower than the ILM group (*P* < 0.05) (Figure 4B). At the genus level, the relative abundances of *Bacteroides, Haemophilus, Actinomyces, Porphyromonas, Gardnerella*, and *Gemella* in the NHM group were significantly lower than the NLM group (*P* < 0.05 or *P* < 0.001 or *P* < 0.0001), and the relative abundances of *Klebsiella* and *Helicobacter* in the NHM group were significantly higher than the NLM group (*P* < 0.05 or *P* < 0.0001) (Figure 4C). The relative abundances of *Bifidobacterium, Streptococcus*, and *Sphingomonas* in the IHM group were significantly lower than that in the ILM group (*P* < 0.05 or *P* < 0.001), and the relative abundances of *Fusobacterium, Coprococcus, [Ruminococcus]_torques_group, Dorea*, and *Butyricicoccus* in the IHM group were significantly higher than the ILM group (*P* < 0.05) (Figure 4D).

To determine the discriminant power of microbiota signatures under the environmental state of microplastic exposure, we applied random forest analysis. Based on the analysis of nasal microbiota, the abscissa of species is positively correlated with the environmental state of microplastic exposure, such as *Klebsiella, Actinomyces, Haemophilus, Ralstonia, Bacteroides*, and *Acinetobacter*, featured as microbiota members for the discrimination between groups (Supplementary Figure 3A). Based on the analysis of intestinal microbiota, the abscissa of species is positively correlated with the environmental state of microplastic exposure, such as *Bifidobacterium, Fusobacterium, Faecalibacterium, Lachnoclostridium, Parabacteroides*, and *Ruminococcus*, featured as microbiota members for the discrimination between groups (Supplementary Figure 3B).

Based on the sequence of a marked gene, the PICRUSt2 (2.3.0b0) software was used to predict the gene function of known microbes, and to show the difference between groups. At level 3 of KEGG, pathways from two groups with significant statistical differences ranked from top to the bottom in bar graphs based on the Wilcoxon method, suggesting that exposure to microplastics may lead to differences in Toll and Imd signaling pathways, Autophagy-yeast, and Cardiac muscle contraction in nasal microbiota (Figure 4E), and differences in cardiac muscle degradation, Prion diseases, *Staphylococcus aureus* infection, styrene degradation, biosynthesis of unsaturated fatty acids, and nitrotoluene in intestinal microbiota (Figure 4F).

### Correlation analysis between the nasal and intestinal microbiota

At the genus level, species from the four groups which were abundance-ranked top 10 were compared based on the Kruskal Wallis method. The results showed that the difference in the abundance of nasal and intestinal shared microbiota was similar in both the low-exposure and high-exposure groups. For example, *Corynebacterium, Staphylococcus, Ralstonia*, and *Lawsonella* were more abundant in the intestine than the nasal cavity, while *Bacteroides, Prevotella, Faecalibacterium, Alistipes*, and *Escherichia-Shigella* were less abundant in the intestine than nasal cavity (*P* < 0.05 or *P* < 0.01 or *P* < 0.001) (Supplementary Figure 4).

**Figure 5:**
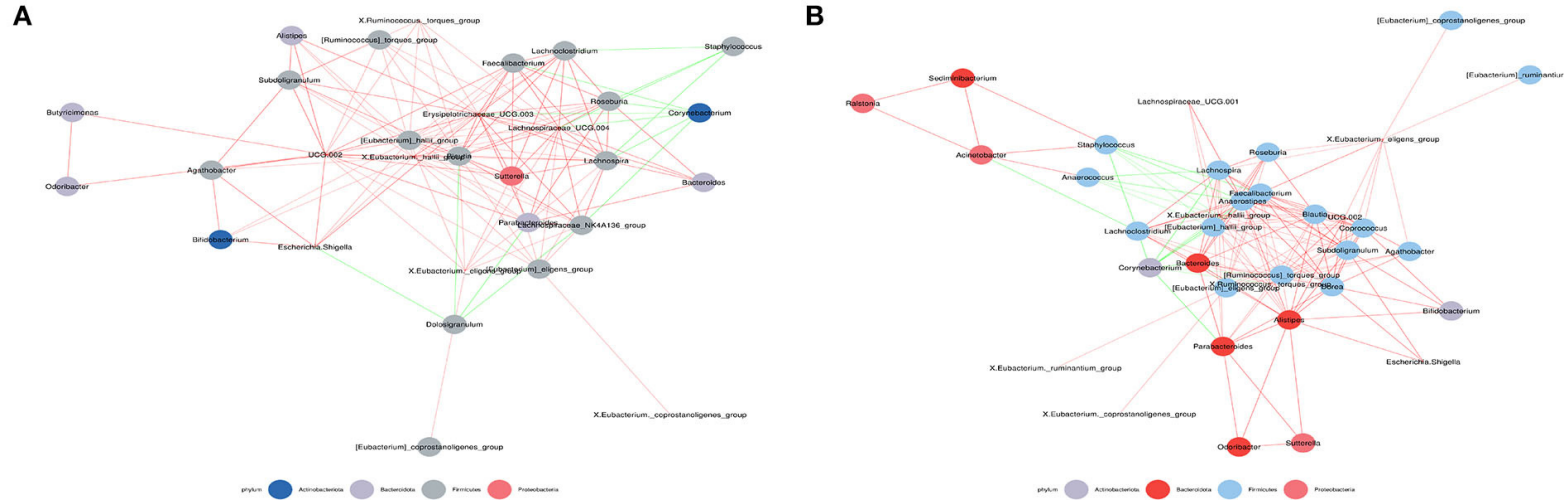
Co-occurrence network map. **(A)** Correlation analysis of intestinal and nasal microbiota at low-exposure group. **(B)** Correlation analysis of intestinal and nasal microbiota at high-exposure group. Species with Spearman Coef > 0.8 and *p* < 0.01 are shown by default in the figure. The size of nodes indicates the abundance of species, and different colors indicate different species. The color of the line indicates a positive and negative correlation, red indicates positive correlation, and green indicates negative correlation. The thickness of the line indicates the Pearson correlation coefficient, the thicker the line is, the more correlative species are. The more lines, the more connected one species is to others.

The symbiotic relationship among microbes is significantly different in the nasal cavity and gut. Therefore, spearman correlation coefficient calculation was performed based on the relative abundances among species to obtain the network of interrelationships of species (Figure 5). Analysis for the abundance-ranked top 50 species at the genus level suggests that exposure to microplastics may lead to alterations in the correlation between intestinal and nasal microbiota in subjects. For example, *Corynebacterium* was not correlated with *Bacteroides* in the low-exposure group, but negatively correlated in the high-exposure group; *Corynebacterium* was not correlated with *X. Ruminococcus.torquespes* in the low-exposure group, but negatively correlated with the high-exposure group; *Staphylococcus* was not correlated with *X. Ruminococcus torquespes* at low-exposure group, but negatively correlated at-high exposure group; *Alisbacterium* was not correlated with *Bifidobacterium* at the low-exposure group, but positively correlated at high-exposure group; *Alistioides* was not correlated with *Bacteroides* at the low-exposure group, but positively correlated at high-exposure group, et cetera.

Indicator analysis revealed the indicator species for each group. The bubble size is positively correlated with the indicator value of each species in this group. The results showed that exposure to microplastics may not only lead to alterations in the abundance of intestinal and nasal microbiota but also cause changes in the correlation between intestinal and nasal microbiota, which is consistent with the conclusion of network analysis (Supplementary Figure 5).

### Correlation analysis of microplastics and microbiota in intestinal secretions

**Figure 6:**
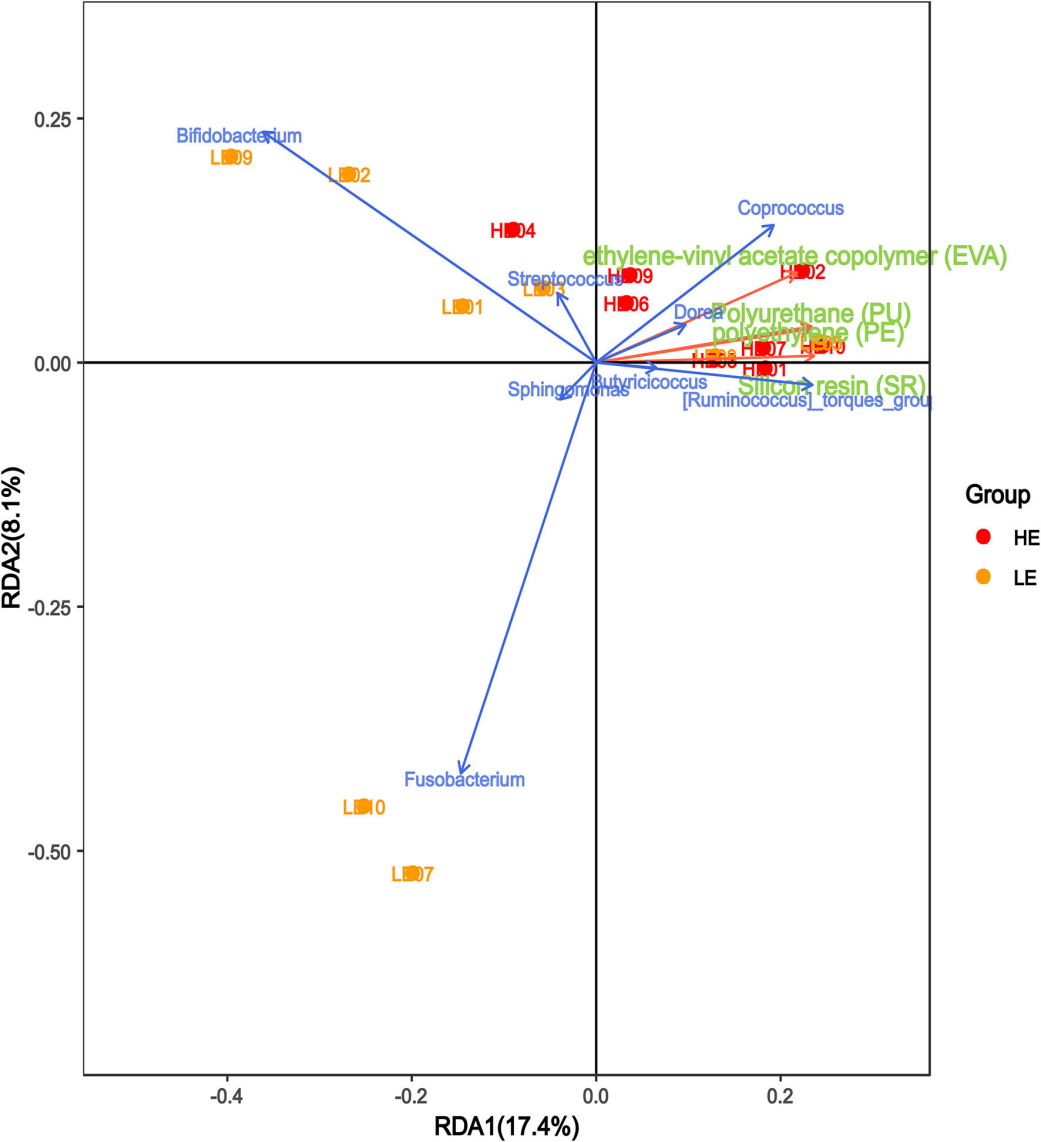
Correlation analysis of microplastics and microbiota in intestinal secretions. The red arrows in the figure represent different microplastic components, and the blue arrows represent different flora. The angle between the flora and microplastic components represents the positive and negative correlation between the two. Acute angles, positive correlation; obtuse angles, negative correlation; right angles, no correlation.

We performed canonical correspondence analysis between the bacterial species within the genera and the main microplastic components of intestinal secretions to reflect the relationship between the flora and the environment. As shown in Figure 6, the microplastic composition was positively correlated with the HM group and negatively correlated with the LM group. The bacterial species within the genera *Coprococcus, Dorea, [Ruminococcus]_torques_group*, and *Butyricicoccus* were positively correlated with microplastic composition, while *Bifidobacterium, Streptococcus, Sphingomonas*, and *Fusobacterium* were negatively correlated with microplastic composition.

## Discussion

In this study, air and soil samples from microplastic low-exposure (Huanhuaxi Park) and high-exposure areas (plastic factories) were examined. The detected points of microplastics in the high-exposure area were significantly more than the low-exposure area. Among them, polyurethane (PU) was the main microplastic component detected, which was the main plastic product in the plastic factory.

Nasal and intestinal secretions were collected from 40 subjects (20:20) who have been working and living in low-exposure and high-exposure areas to microplastics. Specific effort was taken to preserve the microbiota by flash-freezing of nasal and intestinal secretions, to avoid any technical confounders. The results of the examination showed that the microplastic content of intestinal secretions was significantly higher in the high-exposure group than in the low-exposure group. Among them, polyurethane (PU), silicone resin (SR), ethylene-vinyl acetate copolymer (EVA), and polyethylene (PE) were significantly higher in the high-exposure group than the low-exposure group. Furthermore, the bacterial species within the genera *Coprococcus, Dorea, [Ruminococcus]_torques_group*, and *Butyricicoccus* were positively correlated with these microplastics, while *Bifidobacterium, Streptococcus, Sphingomonas*, and *Fusobacterium* were negatively correlated with these microplastics.

Microbiological analysis from 40 subjects showed that nasal and intestinal microbiota shared ASV, which may be due to swallowed snot. At the phylum level, *Bacteroidota* and *Firmicutes* were the dominant strains in the intestinal microbiota, and *Actinobacteriota* and *Firmicutes* were the dominant strains in the nasal microbiota. At the genus level, *Bacteroides* and *Prevotella* were the dominant strains in the intestinal microbiota, and *Corynebacterium* and *Staphylococcus* were the dominant microbiota in the nasal microbiota. The dominant microbiota between the nasal cavity and intestine in the same group were significantly different, in line with the objective differences in colonization areas.

Studies have shown that *bacteroides* have antioxidant effects and beneficial effects on the body (28). Clinical studies have found that the abundance of *bacteroides* decreases in patients with allergic airway diseases (29, 30). *Klebsiella* is closely related to the occurrence of chronic sinusitis and chronic diseases of the upper airways (31, 32). *Helicobacter* is a microaerophilic Gram-negative spirochete that has been proven to be a causative factor for gastric ulcers, gastritis, and gastric cancer (33). *Helicobacter* can colonize not only the gastric mucosa but also the nasal mucosa due to indirect contact infection (34). We found that the relative abundances of *Bacteroides* in the NHM group were significantly lower than in the NLM group. Simultaneously, the relative abundances of *Klebsiella* and *Helicobacter* in the NHM group were significantly higher than the NLM group. These results suggested that exposure to microplastics may increase the abundance of nasal microbiota that are positively associated with respiratory and digestive tract diseases, while reducing the abundance of beneficial microbiota in the nasal cavity.

Studies have found that the bacterial species within the genera *Bifidobacterium, Streptococcus*, are known to possess gluten probiotic properties and the ability to produce SCFA (35), which has an important role as a probiotic in the prevention and treatment of intestinal and parenteral diseases (36, 37). Moreover, *Bifidobacterium* also has anti-IL-6, anti-inflammatory, as well as immune-enhancing effects (38). It can conduct biodegradation and destroy microplastics through its enzymatic hydrogen peroxide activation under semi-anaerobic conditions, thus predicting high exposure of microplastics in gastrointestinal systems with low *Bifidobacterium* levels (39). The abundance of *Streptococcus* and *Sphingomonas* is negatively correlated with the occurrence of colorectal cancer (40, 41). The abundance of *Sphingomonas* decreased in patients with gastric inflammation (42). The results of clinical studies showed that *[Ruminococcus]_torques_group* and *Dorea* in the intestine of patients with irritable bowel syndrome were significantly higher than the healthy controls (43). *Fusobacterium* can cause opportunistic infections that are clearly associated with inflammatory bowel disease and colorectal cancer (44, 45). The abundance of *Coprococcus* is associated with diarrhea, intestinal salmonella infection, and liver tumors (46, 47). We found that the relative abundances of *Bifidobacterium*, Streptococcus, and *Sphingomonas* in the IHM group were significantly lower than that in the ILM group. Simultaneously, the relative abundances of *[Ruminococcus]_torques_group, Dorea, Fusobacterium*, and *Coprococcus* in the IHM group were significantly higher than the ILM group. These results suggested that exposure to microplastics may increase the abundance of intestinal microbiota that are positively associated with digestive tract diseases, while reducing the abundance of beneficial microbiota in the gut.

PICRUSt2 (2.3.0b0) would be used to annotate the gene function of known microbes. The results showed that there were differences in intestinal microbiota in Styrene and Nitrotoluene degradation pathways, indicating that under the environmental state of microplastic exposure, there was a more active microplastic metabolism in intestinal microbiota in the high-exposure group.

The symbiotic relationship among microbes is significantly different between the nasal cavity and the gut. The combined analysis revealed that exposure to microplastics may not only lead to changes in dominant microbiota in the intestine and nasal cavity but also cause correlation changes of nasal and intestinal shared microbiota, such as *Corynebacterium-Bacteroides, Alistipes-Bacterquesoides, Alistipes-Bifidobacterium, Coryneum-X.Ruminococcus.torques group*, Staphylocus-X.Ruminococcus.torocc group, etc.

However, there are still some limitations of this study. Participants in the study group were all from one facility, which may not be representative of the entire high-exposure population. However, it is an indisputable fact that the subjects involved in this study were exposed to microplastics, and the results of the study can reflect the impact of microplastics (especially polyurethane) exposure on human intestinal and nasal microbes to a certain extent.

Overall, the results innovatively revealed how microplastics can affect intestinal and nasal microecosystem. Hopefully, the study will provide reliable evidence to answer the question that how microplastics can affect human health.

## Data availability statement

The data presented in the study are deposited in the NCBI SRA database, accession number: https://dataview.ncbi.nlm.nih.gov/object/SAMN30121005.

## Ethics statement

The studies involving human participants were reviewed and approved by the Medical Ethics Committee of Hospital of Chengdu University of Traditional Chinese Medicine. The patients/participants provided their written informed consent to participate in this study. Written informed consent was obtained from the individual(s) for the publication of any potentially identifiable images or data included in this article.

## Author contributions

CX and YL conceived and designed the experiments. XZ, SP, and YH carried out the experiments. XZ, RT, and YX analyzed the data. JK and HY provided advice. XZ and HW wrote the manuscript. All authors approved the final version of the manuscript.

## Funding

This study was supported by the Chengdu University of Traditional Chinese Medicine Xinglin Scholars Scientific Research Fund (BSH2019024) and Sichuan Provincial Scientific and Technological Innovation Seedling Project (2022036).

## Conflict of interest

The authors declare that the research was conducted in the absence of any commercial or financial relationships that could be construed as a potential conflict of interest.

## Publisher's note

All claims expressed in this article are solely those of the authors and do not necessarily represent those of their affiliated organizations, or those of the publisher, the editors and the reviewers. Any product that may be evaluated in this article, or claim that may be made by its manufacturer, is not guaranteed or endorsed by the publisher.

## Supplementary material

Supplementary Figure 1 Schematic diagram of environmental specimen collection.

Supplementary Figure 2 The laser infrared imaging diagrams of the two groups.

Supplementary Figure 3 Random forest analysis. Abscissa MeanDecreaseGini is the measure of importance, ordinate represents species. The value of MeanDecreaseGini is positively correlated with the reliability of one species as biomarker screening between groups. **(A)** The nasal microbiota. **(B)** The intestinal microbiota.

Supplementary Figure 4 Kruskal Wallis analysis.

Supplementary Figure 5 Indicator analysis. The histogram represents the relative abundance of each ASV.

Supplementary Figure 6 Rarefaction curve.

Supplementary Datasheet 1 Error rate.

Supplementary Datasheet 2 The features of sequencing runs.

Supplementary Datasheet 3 The representative read quality generated by DADA2.

Supplementary Datasheet 4 q-value (FDR-P) and *p*-value.

## References

[1] Thompson RC Olsen Y Mitchell RP Davis A Rowland SJ John AW. Lost at sea: where is all the plastic? Science. (2004) 304:838. 10.1126/science.1094559 15131299

[2] Weinstein JE Crocker BK Gray AD. From macroplastic to microplastic: Degradation of high-density polyethylene, polypropylene, and polystyrene in a salt marsh habitat. Environ Toxicol Chem. (2016) 35:1632–40. 10.1002/etc.3432 26992845

[3] Barnes DK Galgani F Thompson RC Barlaz M. Accumulation and fragmentation of plastic debris in global environments. Philosophical transactions of the royal society of London series B. Biol Sci. (2009) 364:1985–98. 10.1098/rstb.2008.0205 19528051 PMC2873009

[4] Danopoulos E Jenner LC Twiddy M Rotchell JM. Microplastic Contamination of seafood intended for human consumption: a systematic review and meta-analysis. Environ Health Perspect. (2020) 128:126002. 10.1289/EHP7171 33355482 PMC7757379

[5] Eriksen M Lebreton LC Carson HS Thiel M Moore CJ Borerro JC. Plastic pollution in the world's oceans: more than 5 trillion plastic pieces weighing over 250,000 tons afloat at sea. PLoS ONE. (2014) 9:e111913. 10.1371/journal.pone.0111913 25494041 PMC4262196

[6] Toussaint B Raffael B Angers-Loustau A Gilliland D Kestens V Petrillo M. Review of micro- and nanoplastic contamination in the food chain. Food Add Contam Part A Chem Anal Cont Exp Risk Assess. (2019) 36:639–73. 10.1080/19440049.2019.1583381 30985273

[7] Waring RH Harris RM Mitchell SC. Plastic contamination of the food chain: a threat to human health? Maturitas. (2018) 115:64–8. 10.1016/j.maturitas.2018.06.010 30049349

[8] Guo JJ Huang XP Xiang L Wang YZ Li YW Li H. Source, migration, and toxicology of microplastics in soil. Environ Int. (2020) 137:105263. 10.1016/j.envint.2019.105263 32087481

[9] Bessa F Ratcliffe N Otero V Sobral P Marques JC Waluda CM. Microplastics in Gentoo penguins from the Antarctic region. Sci Rep. (2019) 9:14191. 10.1038/s41598-019-50621-2 31578393 PMC6775258

[10] Hirt N Body-Malapel M. Immunotoxicity and intestinal effects of nano- and microplastics: a review of the literature. Part Fibre Toxicol. (2020) 17:57. 10.1186/s12989-020-00387-7 33183327 PMC7661204

[11] Pauly JL Stegmeier SJ Allaart HA Cheney RT Zhang PJ Mayer AG. Inhaled cellulosic and plastic fibers found in human lung tissue. Cancer epidemiology, biomarkers and prevention: a publication of the American association for cancer research, cosponsored by the American society of preventive. Oncology. (1998) 7:419–28. 9610792

[12] Prata JC. Airborne microplastics: consequences to human health? Environ Poll. (2018) 234:115–26. 10.1016/j.envpol.2017.11.043 29172041

[13] Turcotte SE Chee A Walsh R Grant FC Liss GM Boag A. Flock worker's lung disease: natural history of cases and exposed workers in Kingston, Ontario. Chest. (2013) 143:1642–8. 10.1378/chest.12-0920 23699830

[14] Kern DG Kuhn C Ely EW III Pransky GS Mello CJ Fraire AE. Flock worker's lung: broadening the spectrum of clinicopathology, narrowing the spectrum of suspected etiologies. Chest. (2000) 117:251–9. 10.1378/chest.117.1.251 10631226

[15] Atis S Tutluoglu B Levent E Ozturk C Tunaci A Sahin K. The respiratory effects of occupational polypropylene flock exposure. Eur Respir J. (2005) 25:110–7. 10.1183/09031936.04.00138403 15640331

[16] Deng Y Zhang Y Lemos B Ren H. Tissue accumulation of microplastics in mice and biomarker responses suggest widespread health risks of exposure. Sci Rep. (2017) 7:46687. 10.1038/srep46687 28436478 PMC5402289

[17] Deng Y Zhang Y Qiao R Bonilla MM Yang X Ren H. Evidence that microplastics aggravate the toxicity of organophosphorus flame retardants in mice (Mus musculus). J Hazard Mater. (2018) 357:348–54. 10.1016/j.jhazmat.2018.06.017 29908513

[18] Jin Y Lu L Tu W Luo T Fu Z. Impacts of polystyrene microplastic on the gut barrier, microbiota and metabolism of mice. Sci Total Environ. (2019) 649:308–17. 10.1016/j.scitotenv.2018.08.353 30176444

[19] Qiao R Deng Y Zhang S Wolosker MB Zhu Q Ren H. Accumulation of different shapes of microplastics initiates intestinal injury and gut microbiota dysbiosis in the gut of zebrafish. Chemosphere. (2019) 236:124334. 10.1016/j.chemosphere.2019.07.065 31310986

[20] Jin Y Xia J Pan Z Yang J Wang W Fu Z. Polystyrene microplastics induce microbiota dysbiosis and inflammation in the gut of adult zebrafish. Environ Poll. (2018) 235:322–9. 10.1016/j.envpol.2017.12.088 29304465

[21] Liu Z Yu P Cai M Wu D Zhang M Chen M. Effects of microplastics on the innate immunity and intestinal microflora of juvenile Eriocheir sinensis. Sci Total Environ. (2019) 685:836–46. 10.1016/j.scitotenv.2019.06.265 31247433

[22] Ju H Zhu D Qiao M. Effects of polyethylene microplastics on the gut microbial community, reproduction and avoidance behaviors of the soil springtail, Folsomia candida. Environ Poll. (2019) 247:890–7. 10.1016/j.envpol.2019.01.097 30735918

[23] Zhang X He Y Xie Z Peng S Xie C Wang H. Effect of microplastics on nasal and gut microbiota of high-exposure population: protocol for an observational cross-sectional study. Medicine. (2022) 101:e30215. 10.1097/MD.0000000000030215 36042641 PMC9410575

[24] Nossa CW Oberdorf WE Yang L Aas JA Paster BJ Desantis TZ. Design of 16S rRNA gene primers for 454 pyrosequencing of the human foregut microbiome. World journal of gastroenterology. (2010) 16:4135–44. 10.3748/wjg.v16.i33.4135 20806429 PMC2932916

[25] Callahan BJ McMurdie PJ Rosen MJ Han AW Johnson AJ Holmes SP. DADA2: High-resolution sample inference from Illumina amplicon data. Nat Methods. (2016) 13:581–3. 10.1038/nmeth.3869 27214047 PMC4927377

[26] Bolyen E Rideout JR Dillon MR Bokulich NA Abnet CC Al-Ghalith GA. Reproducible, interactive, scalable and extensible microbiome data science using QIIME 2. Nat Biotechnol. (2019) 37:852–7. 10.1038/s41587-019-0209-9 31341288 PMC7015180

[27] Kim BR Shin J Guevarra R Lee JH Kim DW Seol KH. Deciphering Diversity Indices for a Better Understanding of Microbial Communities. J Microbiol Biotechnol. (2017) 27:2089–93. 10.4014/jmb.1709.09027 29032640

[28] Wu C Jeong MY Kim JY Lee G Kim JS Cheong YE. Activation of ectopic olfactory receptor 544 induces GLP-1 secretion and regulates gut inflammation. Gut Microbes. (2021) 13:1987782. 10.1080/19490976.2021.1987782 34674602 PMC8632334

[29] Pang W Jiang Y Li A Zhang J Chen M Hu L. Bacteroides thetaiotaomicron ameliorates experimental allergic airway inflammation via activation of ICOS(+)Tregs and inhibition of Th2 response. Front Immunol. (2021) 12:620943. 10.3389/fimmu.2021.620943 33815374 PMC8010693

[30] Ouwehand AC Nermes M Collado MC Rautonen N Salminen S Isolauri E. Specific probiotics alleviate allergic rhinitis during the birch pollen season. World journal of gastroenterology. (2009) 15:3261–8. 10.3748/wjg.15.3261 19598302 PMC2710782

[31] Kim D Assiri AM Kim JH. Recent trends in bacteriology of adult patients with chronic rhinosinusitis. J Clin Med. (2019) 8:1889. 10.3390/jcm8111889 31698781 PMC6912634

[32] Botelho-Nevers E Gouriet F Lepidi H Couvret A Amphoux B Dessi P. Chronic nasal infection caused by Klebsiella rhinoscleromatis or Klebsiella ozaenae: two forgotten infectious diseases. Int Soc Infect Dis. (2007) 11:423–9. 10.1016/j.ijid.2006.10.005 17337224

[33] Salama NR. Cell morphology as a virulence determinant: lessons from Helicobacter pylori. Curr Opin Microbiol. (2020) 54:11–7. 10.1016/j.mib.2019.12.002 32014717 PMC7247928

[34] Kumral TL Gökden Y Saltürk Z Berkiten G Yildirim G Ataç E. The effect of gastric helicobacter pylori colonization on nasal functions. Ear Nose Throat J. (2019) 98:346–50. 10.1177/0145561319840825 31018689

[35] Bodkhe R Shetty SA Dhotre DP Verma AK Bhatia K Mishra A. Comparison of small gut and whole gut microbiota of first-degree relatives with adult celiac disease patients and controls. Front Microbiol. (2019) 10:164. 10.3389/fmicb.2019.00164 30800106 PMC6376745

[36] Hidalgo-Cantabrana C Delgado S Ruiz L Ruas-Madiedo P Sánchez B Margolles A. Bifidobacteria and their health-promoting effects. Microbiol Spectrum. (2017) 5:1128. 10.1128/microbiolspec.BAD-0010-2016 28643627 PMC11687494

[37] Engevik MA Luk B Chang-Graham AL Hall A Herrmann B Ruan W. Bifidobacterium dentium fortifies the intestinal mucus layer via autophagy and calcium signaling pathways. mBio. (2019) 10:19. 10.1128/mBio.01087-19 31213556 PMC6581858

[38] Bozkurt HS Bilen Ö. Oral booster probiotic bifidobacteria in SARS-CoV-2 patients. Int J Immunopathol Pharmacol. (2021) 35:20587384211059677. 10.1177/20587384211059677 34818923 PMC8649091

[39] Bozkurt HS Yörüklü HC Bozkurt K Denktaş C Bozdogan A Özdemir O. Biodegradation of microplastic by probiotic bifidobacterium. Int J Glob Warm. (2022) 26:122435. 10.1504/IJGW.2022.122435

[40] Kim DJ Yang J Seo H Lee WH Ho Lee D Kym S. Colorectal cancer diagnostic model utilizing metagenomic and metabolomic data of stool microbial extracellular vesicles. Sci Rep. (2020) 10:2860. 10.1038/s41598-020-59529-8 32071370 PMC7029032

[41] Clos-Garcia M Garcia K Alonso C Iruarrizaga-Lejarreta M D'Amato M Crespo A. Integrative analysis of fecal metagenomics and metabolomics in colorectal cancer. Cancers. (2020) 12:24. 10.2139/ssrn.3520024 32370168 PMC7281174

[42] Sung JJY Coker OO Chu E Szeto CH Luk STY Lau HCH. Gastric microbes associated with gastric inflammation, atrophy and intestinal metaplasia 1 year after Helicobacter pylori eradication. Gut. (2020) 69:1572–80. 10.1136/gutjnl-2019-319826 31974133 PMC7456733

[43] Yang J Wang P Liu T Lin L Li L Kou G. Involvement of mucosal flora and enterochromaffin cells of the caecum and descending colon in diarrhoea-predominant irritable bowel syndrome. BMC Microbiol. (2021) 21:316. 10.1186/s12866-021-02380-2 34773967 PMC8590216

[44] Brennan CA Garrett WS. Fusobacterium nucleatum - symbiont, opportunist, and oncobacterium. Nat Rev Microbiol. (2019) 17:156–66. 10.1038/s41579-018-0129-6 30546113 PMC6589823

[45] Engevik MA Danhof HA Ruan W Engevik AC Chang-Graham AL Engevik KA. Fusobacterium nucleatum secretes outer membrane vesicles and promotes intestinal inflammation. mBio. (2021) 12:20. 10.1128/mBio.02706-20 33653893 PMC8092269

[46] Xu X Gong L Wang B Wu Y Wang Y Mei X. Glycyrrhizin attenuates salmonella enterica serovar typhimurium infection: new insights into its protective mechanism. Front Immunol. (2018) 9:2321. 10.3389/fimmu.2018.02321 30459751 PMC6232675

[47] Pedersen KB Pulliam CF Patel A Del Piero F Watanabe TTN Wankhade UD. Liver tumorigenesis is promoted by a high saturated fat diet specifically in male mice and is associated with hepatic expression of the proto-oncogene Agap2 and enrichment of the intestinal microbiome with Coprococcus. Carcinogenesis. (2019) 40:349–59. 10.1093/carcin/bgy141 30325408 PMC6487682

