# Supplementary figures and images for: Effect of microplastics on nasal and intestinal microbiota of the high-exposure population
Source: Front Public Health. 2022 Oct 28;10:1005535. doi: 10.3389/fpubh.2022.1005535 (PMC9650105; doi:10.3389/fpubh.2022.1005535)

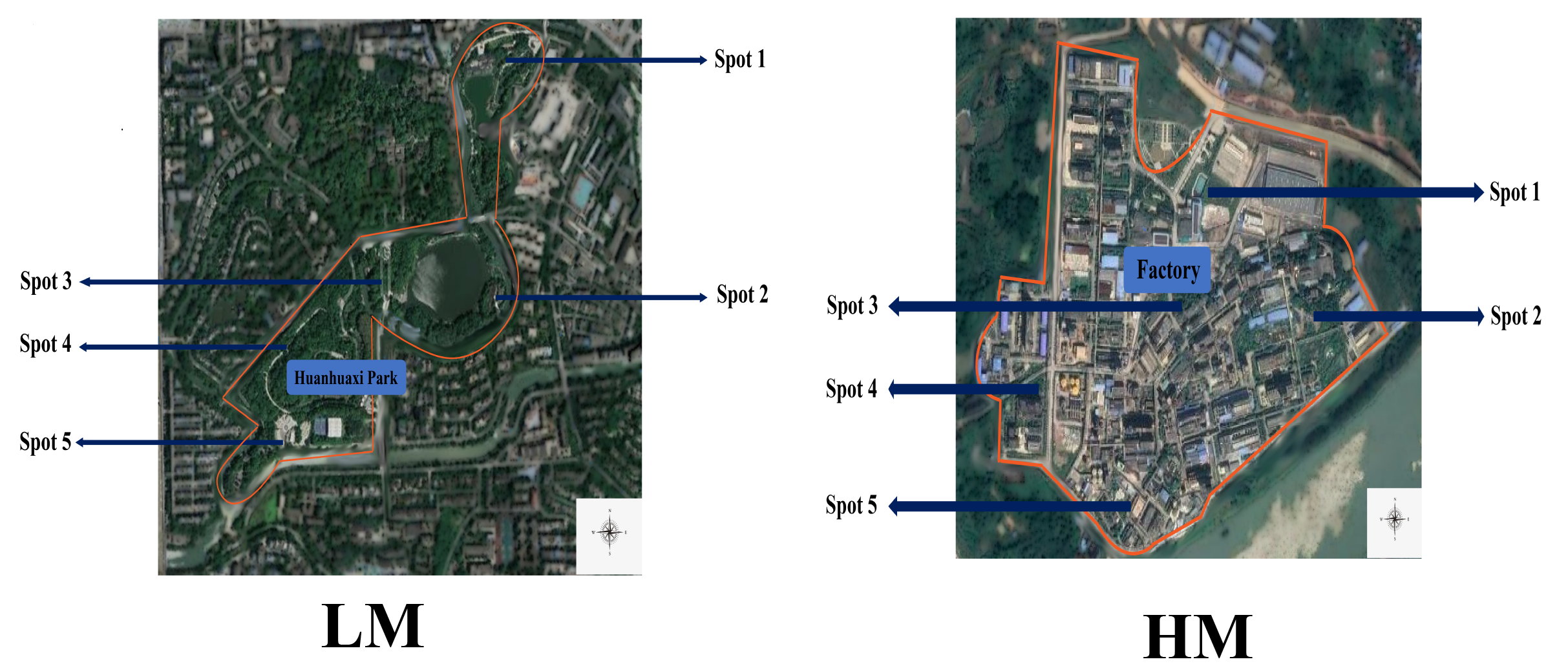

Supplement: Supplementary Figure 1 — Schematic diagram of environmental specimen collection. [file Image_1.TIF]

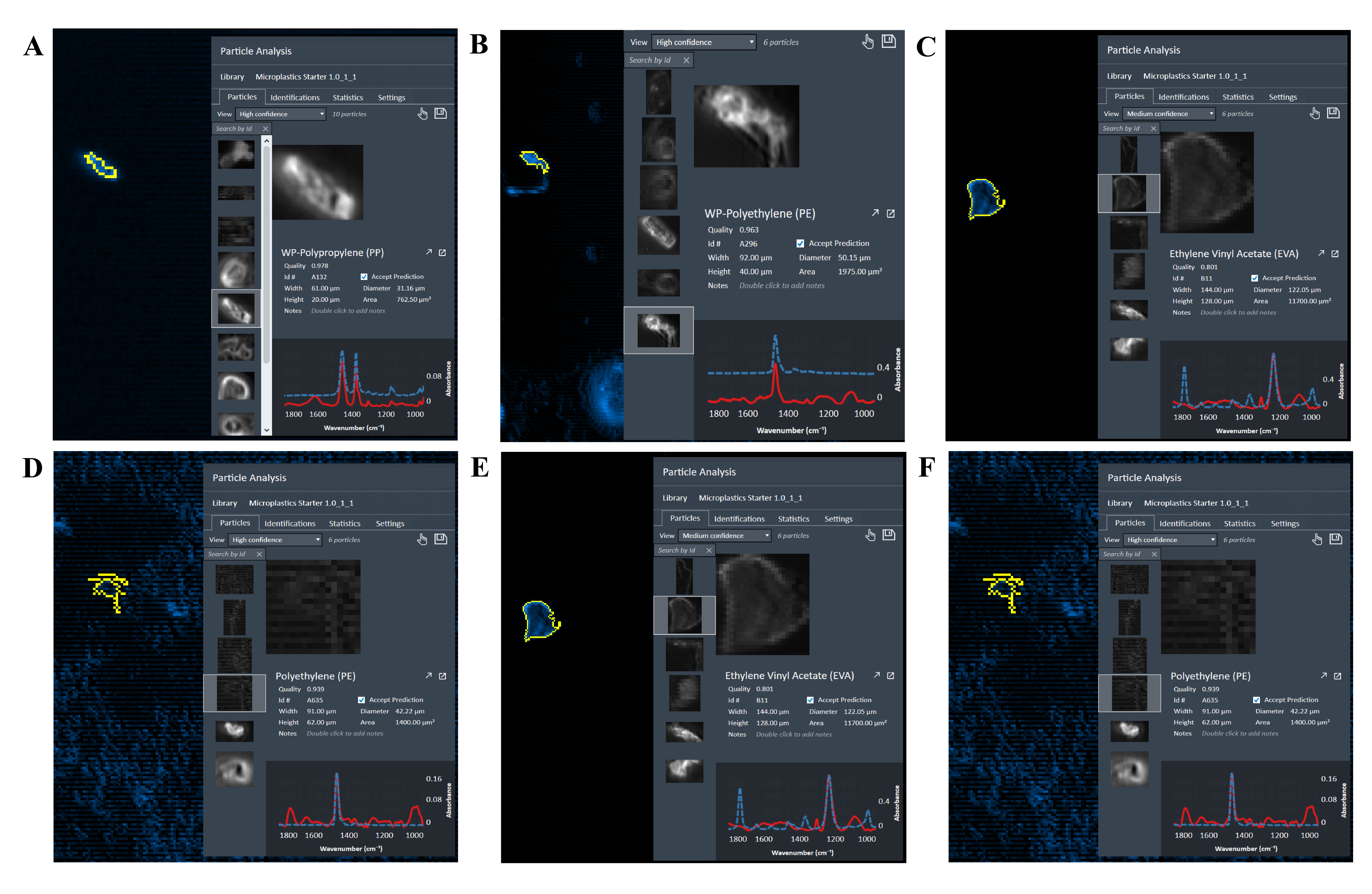

Supplement: Supplementary Figure 2 — The laser infrared imaging diagrams of the two groups. [file Image_2.TIF]

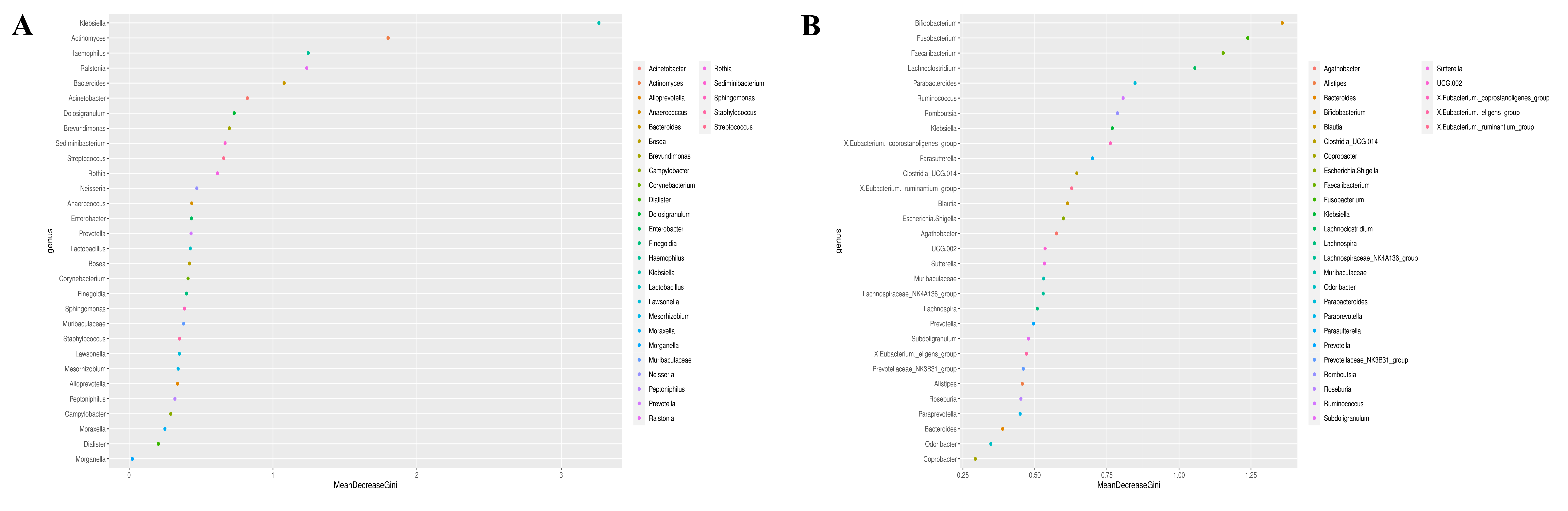

Supplement: Supplementary Figure 3 — Random forest analysis. Abscissa MeanDecreaseGini is the measure of importance, ordinate represents species. The value of MeanDecreaseGini is positively correlated with the reliability of one species as biomarker screening between groups. (A) The nasal microbiota. (B) The intestinal microbiota. [file Image_3.TIF]

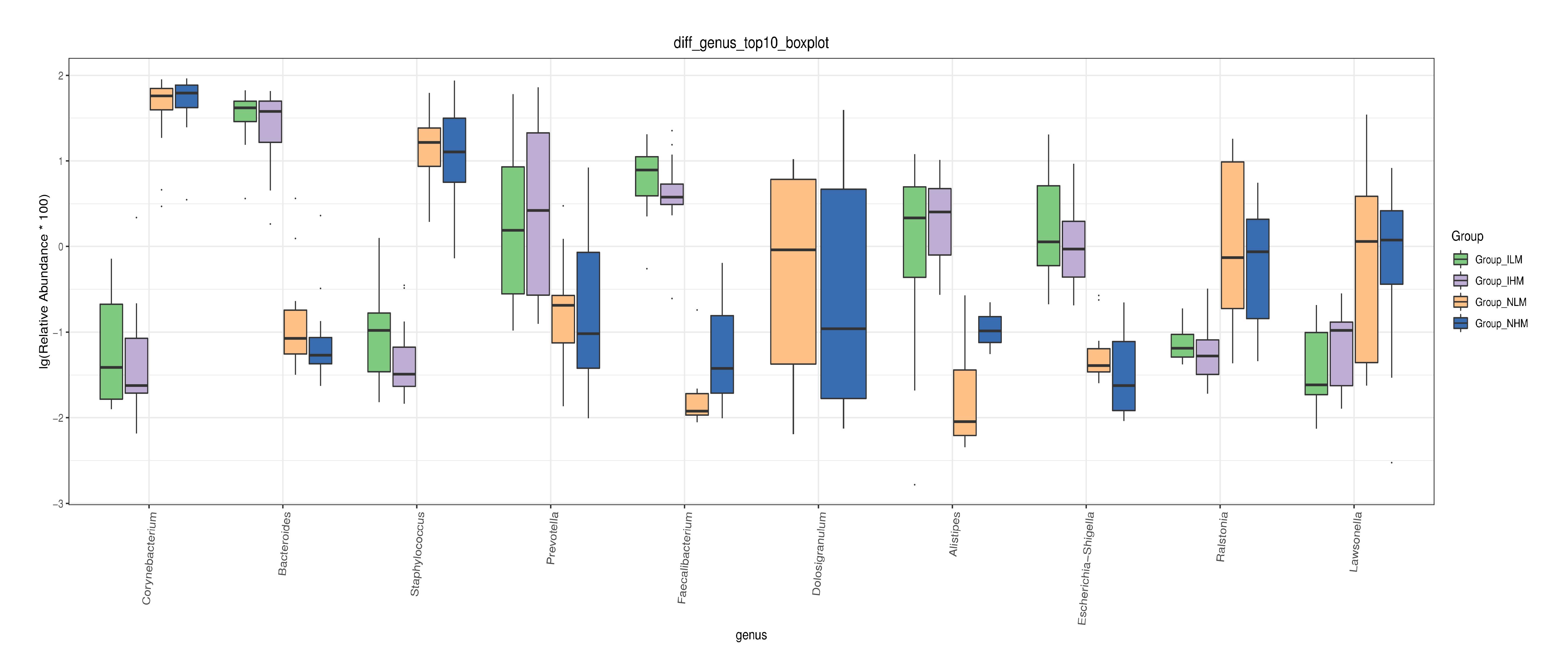

Supplement: Supplementary Figure 4 — Kruskal Wallis analysis. [file Image_4.JPEG]

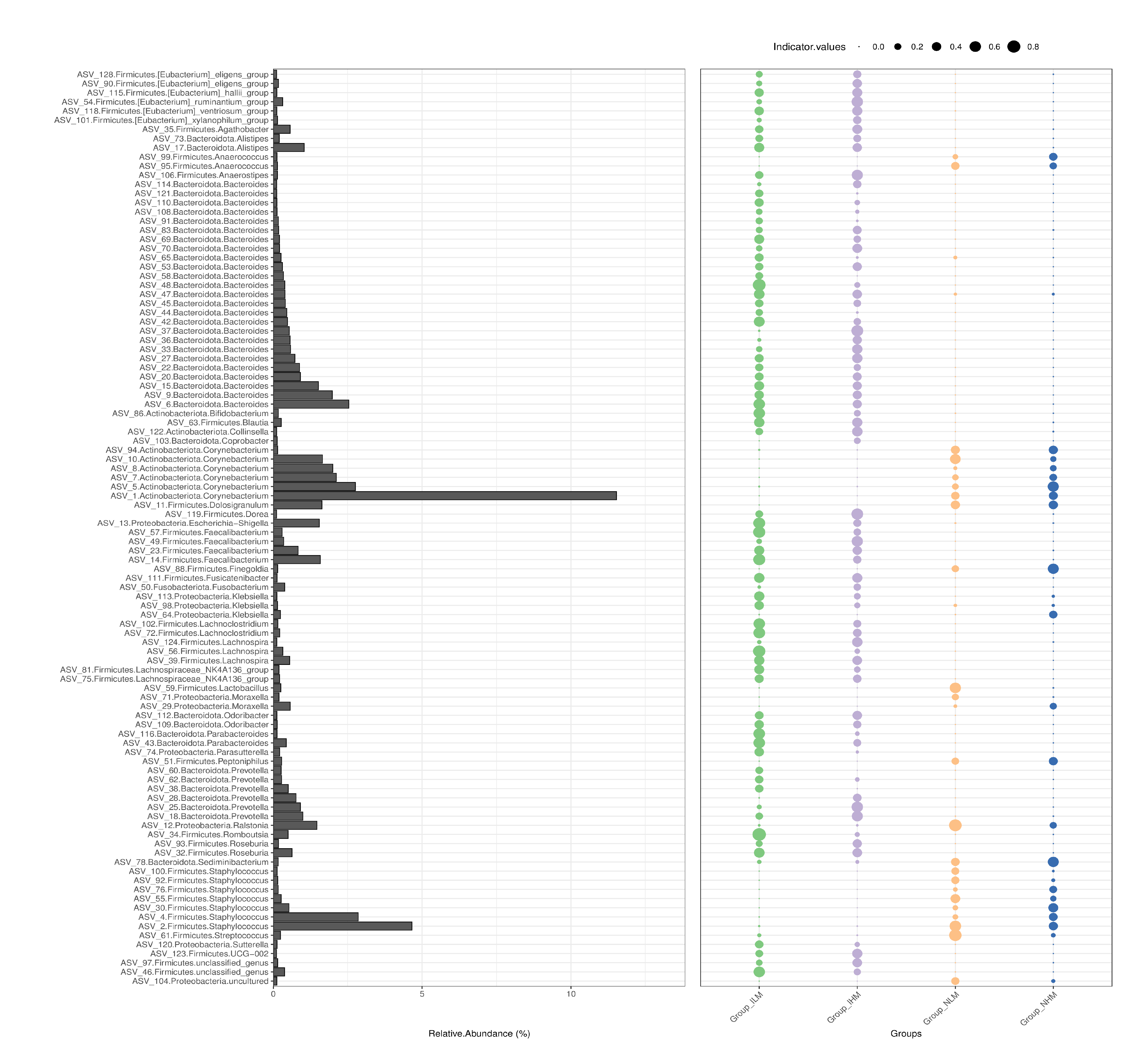

Supplement: Supplementary Figure 5 — Indicator analysis. The histogram represents the relative abundance of each ASV. [file Image_5.TIF]

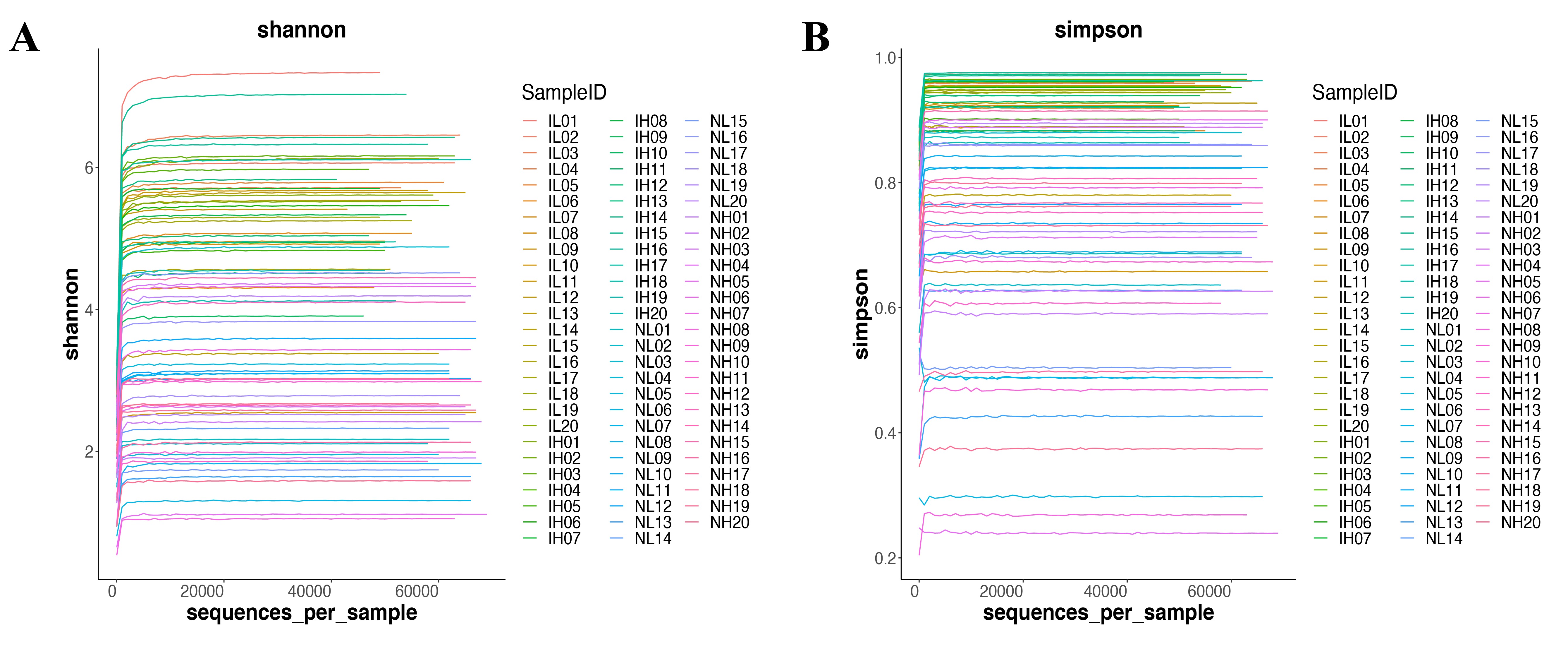

Supplement: Supplementary Figure 6 — Rarefaction curve. [file Image_6.JPEG]
